# Hookworm infection is associated with decreased CD4^+^ T cell counts in HIV-infected adult Ugandans

**DOI:** 10.1371/journal.pntd.0005634

**Published:** 2017-05-25

**Authors:** Bozena M. Morawski, Miya Yunus, Emmanuel Kerukadho, Grace Turyasingura, Logose Barbra, Andrew Mijumbi Ojok, Andrew R. DiNardo, Stefanie Sowinski, David R. Boulware, Rojelio Mejia

**Affiliations:** 1 Division of Epidemiology and Community Health, School of Public Health, University of Minnesota, Minneapolis, MN, United States of America; 2 Division of Infectious Diseases and International Medicine, Department of Medicine, University of Minnesota, Minneapolis, MN, United States of America; 3 The AIDS Support Organization, Kampala, Uganda; 4 Baylor College of Medicine Children's Foundation, Kampala, Uganda; 5 Infectious Diseases Institute, Makerere University, Kampala, Uganda; 6 Division of Global and Immigrant Health, Department of Pediatrics, Baylor College of Medicine, Houston, TX, United States of America; 7 The Gladstone Institute of Virology and Immunology, University of California, San Francisco, San Francisco, CA, United States of America; 8 Section of Tropical Medicine, Department of Pediatrics, National School of Tropical Medicine, Baylor College of Medicine, Houston, TX, United States of America; Swiss Tropical and Public Health Institute, SWITZERLAND

## Abstract

Most studies evaluating epidemiologic relationships between helminths and HIV have been conducted in the pre-ART era, and evidence of the impact of helminth infections on HIV disease progression remains conflicting. Less is known about helminth infection and clinical outcomes in HIV-infected adults receiving antiretroviral therapy (ART). We sampled HIV-infected adults for eight gastrointestinal parasites and correlated parasitic infection with demographic predictors, and clinical and immunologic outcomes. Contrasting with previous studies, we measured parasitic infection with a quantitative, highly sensitive and specific polymerase chain reaction (PCR) method. This cohort study enrolled HIV-infected Ugandans from August-September 2013 in Mbale, Uganda and collected stool and blood samples at enrollment. Real-time PCR quantified stool: *Ascaris lumbricoides*, *Ancylostoma duodenale*, *Necator americanus*, *Strongyloides stercoralis*, *Trichuris trichiura*, *Cryptosporidium spp*., *Entamoeba histolytica*, and *Giardia intestinalis* infection. Generalized linear models assessed relationships between parasitic infection and clinical or demographic data. 35% of participants (71/202) tested positive for ≥1 helminth, mainly *N*. *americanus* (55/199, 28%), and 4.5% (9/202) were infected with ≥2 stool parasites. Participants with hookworm infection had lower average CD4^+^ cell counts (-94 cells/mcL, 95%CI: -141, -48 cells/mcL; p<0.001) after adjustment for sex, CD4^+^ nadir at clinic entry, and time on ART. The high prevalence of parasitic infection and correlation with decreased CD4^+^ concentrations highlight the need to re-examine the effects of invasive helminth co-infection in rural, HIV-infected populations in the era of widely available ART. Elucidating the relationship between hookworm infection and immune recovery could provide opportunities for health optimization, e.g. integrated deworming, in these vulnerable populations.

## Author summary

Intestinal parasites, diseases of poverty that infect low-income populations and decrease school attendance and earning potential, infect more than 1 billion people worldwide. Current international guidelines focus deworming campaigns on high-risk populations of preschool- and school-aged children, and women of childbearing age. Intestinal parasitic infections also overlap significantly with human immunodeficiency virus (HIV), which is highly concentrated in Sub-Saharan Africa; and some research indicates that certain intestinal parasitic infections have contributed to the spread of HIV in Sub-Saharan Africa. While there is a significant body of research examining the intersection between intestinal parasites and HIV disease progression, most of this work has occurred before HIV therapy was widely available in the region. This is the first study, to our knowledge, that focuses on a mixed sex, HIV treatment-initiated adult population, and directly measures intestinal parasitic infection via highly sensitive and specific molecular techniques. The results of this study indicate that adults who are living with HIV and parasitic hookworm infection are at an immunologic disadvantage when compared to adults who are not infected with hookworm. Our results also suggest that integrating deworming medications into HIV care could be an effective way to maximize the health status of this vulnerable population.

## Introduction

Five soil transmitted helminth species *Ascaris lumbricoides*, *Trichuris trichiura*, hookworm species *Necator americanus* and *Ancylostoma duodenale*, and *Strongyloides stercoralis* infect over a billion people worldwide.[1, 2] The burden of parasitic infection is greatest in low-income areas, particularly in certain areas of sub-Saharan Africa, where human immunodeficiency virus (HIV) is also highly prevalent. Studies of African adults living with HIV have shown helminth co-infection rates that range from 10% to upwards of 45%.[3–9]

To date, the majority of research investigating the impact of intestinal helminth infection on HIV disease progression has occurred prior to widely available antiretroviral therapy (ART).[3, 4, 9–13] The effect of helminth and HIV co-infection in the presence of ART is less well characterized. Indeed, to our knowledge, only two studies to date have examined the impact of deworming on CD4^**+**^ recovery in persons receiving ART.[14, 15] The current literature examining the relationship between soil transmitted helminth infections and HIV in the pre-ART era presents an inconclusive picture. The large body of observational data is mixed. Two observational cohort studies found no beneficial effect of deworming on HIV viral loads and CD4^**+**^ T-cell concentrations,[4, 5] while another suggested the possibility of a protective effect of helminths on decreasing HIV viral replication.[3]

Of the three randomized experiments evaluating the impact of deworming on markers of HIV disease progression without ART, two found an improvement in either CD4^**+**^ T-cell concentrations or HIV viral load after anthelmintic therapy.[9, 13] Another larger, reflexive randomized deworming trial failed to show a statistically significant benefit of empiric deworming treatment versus standard of care in preventing HIV progression to either a CD4^**+**^ count of <350 cells/mcL, first reported use of antiretroviral treatment, or death due to a non-traumatic cause (44.0 versus 49.8 events per 100 person-years; hazard ratio = 0.88, 95%CI 0.74 to 1.04, P = 0.10).[10] However, it is possible that there was a less extreme benefit to presumptive therapy, which they were underpowered to detect.

Interactions between soil-transmitted helminths and their human hosts are complex, and helminth infection may influence a patient’s relationship with other pathogens. A recent review discusses not only the links between selected parasites and HIV susceptibility and disease progression, but also the relationship between soil transmitted helminths and the potential for increased susceptibility to malaria and tuberculosis.[16–18] It is also important to recognize that soil transmitted helminths, through their potent and systemic T helper cell type 2 (Th2) cytokine and regulatory responses,[19] may induce Th2 protective effects that could benefit long-term HIV survivors, e.g. protection against conditions associated with chronic inflammation.[20–23] However, this same Th2 immune response may mediate increased susceptibility for Th1-related infections.[24]

At present, integrated presumptive anthelmintic therapy in the context of HIV care is neither recommended by Ugandan National Guidelines,[25] nor is it recommended by WHO.[26] While WHO does recommend periodic so called “preventive chemotherapy” for high risk groups, including women of child bearing age and adults with occupational exposures,[27] these guidelines have generally not been integrated into any type of standard care, nor has particular emphasis been given to HIV-infection. Given the frequency and consistency with which HIV-diagnosed persons interact with their care providers, the integration of adult deworming programs into HIV care may be a logical conclusion.[28] However, given the dearth of high quality and adequately powered species-specific studies, dramatic increases in ART availability, and incomplete understanding of biological mechanisms that are impacted by helminths during HIV infection implies that research questions focused on soil transmitted helminths and HIV have not been exhausted.

Our current study evaluated the prevalence and burden of the five most common soil-transmitted helminths and three protozoal species in adults living with HIV enrolled in outpatient HIV care in peri-urban Uganda. We also evaluated the relationship between helminth infection and clinical and immunologic outcomes, and examined risk factors for helminth infection in this population.

## Methods

### Ethics statement

Written informed consent was provided by all participants. The University of Minnesota, The AIDS Support Organisation (TASO), and the Uganda National Council of Science and Technology institutional review boards approved this protocol.

### Participant recruitment and data collection

From August through September 2013, we screened HIV-infected adults engaged in outpatient care at the TASO HIV clinic in Mbale, Uganda, during their normal clinical visit for a one-time stool sample analysis, and longitudinal follow-up via chart review. This study was powered to estimate overall parasitic infection prevalence among patients with a recent CD4+ T cell count <500 cells/mcL, a population of approximately 600. We estimated a sample size of 210, based on a true population prevalence of 30%, an alpha level of 0.05, 5% precision estimate, and a finite population size of 600.

Inclusion criteria were age ≥18 and most recent CD4^+^ count <500 cells/mcL. We excluded persons who reported or had a record of taking albendazole or other anthelmintics in the past three months, and persons with known albendazole allergy. Pregnant women were also excluded due to potential albendazole teratogenicity.

Participant data were collected via participant interview and chart review. We collected data on age, sex, weight, village of residence, and occupation. We also collected data on date of HIV diagnosis, date of enrollment into HIV care, World Health Organization (WHO) clinical stage at clinic enrollment, CD4^+^ at enrollment into clinical care (“nadir CD4^+^”), 12-month history of opportunistic infections, and ART history (regimen, duration) through review of medical records by a medical officer. Participants underwent a physical examination at study enrollment for assessment of current WHO clinical stage, weight, and presence of current opportunistic infections. Finally, we collected follow-up CD4^+^ T helper cell values that were gathered as part of TASO’s routine clinical practice in the 24 months since study enrollment. Study follow-up occurred in a passive fashion, and no attempts were made beyond standard clinical practice to return patients to care if they stopped attending clinic.

### Biological sample collection and analyses

We collected blood and stool from participants during their study visit, which was also a participant’s normal clinic visit. We performed a single blood draw to evaluate CD4^+^ T cell count via the FACSCalibur flow cytometer (BD Biosciences, San Jose, CA) per routine TASO laboratory protocol.

Parasitic infection status was only evaluated at one time-point: study enrollment. Participants provided a single stool sample, which we froze without fixatives on site at -20°C within 1–2 hours of collection. Stool specimens were transported on a weekly basis to Kampala, Uganda for long-term -80°C storage during enrollment. At the Translational Research Laboratory of the Infectious Diseases Institute in Kampala, Uganda, we used a modified version of a validated quantitative PCR described previously in Mejia *et al*. to assess participant stool for *Ascaris lumbricoides*, *Ancylostoma duodenale*, *Necator americanus*, *Strongyloides stercoralis*, *Trichuris trichiura*, *Cryptosporidium spp*., *Entamoeba histolytica*, and *Giardia intestinalis* infection.[29] This PCR assay was modified to increase the total volume of each reaction from 7μL to 10μL to accommodate the minimum settings on the Applied Biosystems 7900HT Fast Real-Time PCR System. Reagent concentrations of the 10μL reaction matched those of the 7μL reaction concentrations.[29]DNA was extracted from approximately 50mg of stool via the FastDNA SPIN Kit for Soil (MP Biomedicals, Santa Ana, CA) using a low reagent method developed by Mejia *et al*. for resource-limited contexts, which has been included as S1 DNA Extraction Protocol. An additional step was required to extract *T*. *trichiura* DNA, whereby the remaining insoluble pellet from one DNA extraction was re-suspended in 200μL DNA-free water, heated at 90°C for 10 minutes, and centrifuged at 14,000g for 10 minutes. We then repeated the above-described DNA extraction method to process the resulting soluble portion of the sample.

Sequences for the species-specific primers and probes and methods for the qPCR analysis are found in Mejia *et al*.[29] All control standards were tested in triplicate, and all unknown samples were tested in duplicate. A PCR cycle threshold (C_t_) value >38 was considered a negative result. To help ensure that false positives were not driving our results, we conducted a *post hoc* experiment to bind the *N*. *americanus* primers and probes to the pBR322 internal control plasmid.[30] We did not observe any evidence of binding between the *N*. *americanus* primers or probes and the pBR322 control plasmid.

Parasite burden quantification was performed by interpolating against parasite specific sequences standards and reported as DNA fg/μl.[29, 31] Briefly, egg counts were estimated from McMaster microscopy techniques of subjects infected with *N*. *americanus* and/or *A*. *duodenale* and compared directly to qPCR results. Estimated egg counts from qPCR were calculated using Y_ova/g feces_ = 0.03472*X_fg/μl_ per correlation studies.[31] Similar calculations were used to estimate *Trichuris trichiura* egg counts: Y_ova/g feces_ = (1.095 x 10^−5^)*X_fg/μl_, which was derived by comparing qPCR to Kato-Katz results in infected individuals.[29]

### Statistical analyses

Parasite infection prevalence was estimated overall, by species, and by species type (protozoa or nematoda). Infection intensity was summarized by species for helminth worms.[32, 33] Statistical analyses focused on hookworm infection *a posteriori*, due to its unique immunologic and clinical features, and overwhelming prevalence relative to other species of helminths. We used generalized linear models with a binomial distribution and log link, and a robust covariance estimator, to estimate associations between parasitic infection (overall helminth infection, hookworm infection only, and protozoa infection) and clinical and demographic characteristics, specifically occupation (farming as primary profession versus any other), sex, age (scaled to 5-year increments), weight (scaled to 5-kg increments), WHO Clinical Stage (3 or 4 versus 1 or 2), ART status (receiving or not receiving).

We also estimated the association between parasitic infection and CD4^+^ T cells/mcL at study enrollment, and the potential effect of parasitic infection on over CD4^+^ T cell concentrations over follow-up. Age-, and sex-adjusted linear regression models estimated the mean difference in CD4+ T cells/mcL at study enrollment by parasitic infection status (any protozoa, any helminth, hookworm only). Restricted maximum likelihood linear mixed models, which included participant-specific random intercepts, and an identity covariance matrix, evaluated change in CD4+ T cell concentrations over time across hookworm infection status among participants who were ART-initiated at baseline. These longitudinal models were adjusted for sex, age, time on ART, and weight at baseline. Additional exploratory sub-analyses of change in CD4+ T cell concentrations by hookworm infection status were performed among 1) participants who had initiated ART <1 year before enrollment, and 2) participants who had initiated ART for ≥1 year before enrollment. Time on ART, weight, sex, and age were *a priori* included as covariates given their relationship with either the outcome, or to control for potential confounding, e.g. age.

We attempted to evaluate the relationship between CD4^+^ T cell count and parasite burden (light, moderate, and heavy intensity infections per WHO classification). However, because all infections were classified as light intensity (<2,000 eggs/gram feces), we were unable to create any clinically meaningful exposures beyond presence or absence of hookworm infection. No imputation was performed for missing data, which occurred in <2% of participants. All analyses were performed in Stata/IC 13.1 (StataCorp, College Station, Texas) and results were evaluated against an alpha level of 0.05.

## Results

We consented 216 HIV-infected adults during a routine clinic visit. Of these, 14 potential participants were unable to produce a stool sample on site, and were excluded from the study. Thus, 202 participants were enrolled (**Table 1**). Women comprised 69% of participants (139/202). The participants’ median age was 35 years [IQR: 30, 41]. The median overall CD4^+^ at study enrollment was 375 cells/mcL [IQR: 243, 450], and 90% (181/202) of participants were receiving antiretroviral therapy (ART) for a median duration of 15 (IQR: 5, 29) months. All participants were receiving primary *Pneumocystis jiroveci* pneumonia prophylaxis with either trimethoprim/sulfamethoxazole (n = 201) or dapsone (n = 1).

**Table 1:** Baseline characteristics and demographic information by presence of stool helminth infection.

Characteristic	No helminth infection		Any helminth infection*		P-value
	N	Median [IQR] or n (%)	N	Median [IQR] or n (%)	
Age, years	140	35 [28, 40]	62	36 [30, 43]	0.16
Women	140	90 (64.3%)	62	49 (79.0%)	0.04
Weight, kg	136	53 [47, 60]	60	53 [48, 59]	0.83
CD4^+^ nadir at clinic entry, cells/mcL	135	257 [127, 401]	59	270 [117, 432]	0.50
CD4^+^ at study enrollment visit, cells/mcL	140	390 [280, 467]	62	319 [191, 415]	<0.001
Currently receiving ART	140	129 (92.1%)	62	52 (83.9%)	0.08
Duration of ART, months**	129	15 [5, 28]	52	15 [4, 35]	0.84
Receiving tenofovir**	129	94 (72.9%)	52	33 (63.5%)	0.21
12-month pulmonary tuberculosis history	140	2 (1.4%)	62	1 (1.6%)	0.67
Self-reported farming occupation	139	87 (62.6%)	61	44 (72.1%)	0.19

*Ascaris lumbricoides, *Ancylostoma duodenale*, *Necator americanus*, *Strongyloides stercoralis*, *Trichuris trichiura*

**Among those participants currently receiving ART

### Prevalence & burden

Multi-parallel quantitative PCR results indicated that 35.2% (71/202) of participants were infected with at least one species of helminth or protozoa. Of these 71 participants, 10 were infected with two species. Most parasitic infections were caused by *N*. *americanus* (27.6%, 55/199). *Giardia* had the next highest prevalence (6.1%, 12/197), followed by *Strongyloides* (4.0%, 8/202). Prevalence and infection intensity of parasitic organisms are described in **Table 2**.

**Table 2:** Stool parasite infection and burden by species.

	N	n (%)	DNA (fg/μl) Median [IQR]	Estimated eggs/g stool	WHO Classification
Overall	202	71 (35.2%)	N/A	N/A	
**Helminths**					
*Any helminth infection*	202	62 (30.7%)	N/A	N/A	N/A
*Ascaris lumbricoides*	189	0 (0%)	N/A	N/A	N/A
*Ancylostoma duodenale*	200	1 (1%)	18.3	527	Light
*Necator americanus*	199	55 (27.6%)	0.025 [0.018, 0.22]	0.72 [0.53, 6.34]	Light
*Strongyloides stercoralis*	202	8 (4.0%)	2.1 [<0.1, 81.0]	N/A	N/A
*Trichuris trichiura*	201	1 (0.5%)	0.6	52,694	Heavy
**Protozoa**					
Any protozoa infection*	202	13 (6.4%)	N/A	N/A	N/A
*Cryptosporidium parvum/hominum*	81	1 (1.2%)	35.9 [35.9, 35.9]	N/A	N/A
*Entamoeba histolytica*	201	3 (1.5%)	<0.1 [<0.1, 0.3]	N/A	N/A
*Giardia intestinalis*	197	12 (6.1%)	14.7 [0.3, 205.5]	N/A	N/A

* Infection with either *Entamoeba histolytica* or *Giardia intestinalis*.

Calculated egg burdens for *N*. *americanus* infections had a median of 0.72 eggs per gram of stool (IQR: 0.53, 6.34; maximum: 275) and 527 eggs/gram of stool for the single *Ancylostoma duodenale* infection. These are considered light infections by the World Health Organization.[34] An estimated 52,694 eggs/gram of stool was calculated for the single heavy *Trichuris trichiura* infection. *Strongyloides stercoralis* eggs generally hatch and mostly larvae are seen in stool samples, there are no current categories for intensity of larvae in infected patients.

### Factors associated with protozoal infection

Results of generalized linear models analyses indicated that each 5-year increase in age was inversely related with a composite outcome of either *Giardia*, *Cryptosporidium*, or *E*. *histolytica* infection (Prevalence Ratio (RR) = 0.71, 95%CI: 0.55, 0.92, p = 0.01); 11.5% (6/52) of participants <30 years of age, 6.7% (6/90) of participants 31 to 40 years of age, and 1.7% (1/60) of participants ≥40 years of age were infected with ≥1 protozoal species. Protozoal infection was more prevalent in farmers than other occupations, although this relationship was unstable and not statistically significant in an age- and sex-adjusted model (PR = 3.96; 95%CI: 0.89, 17.60; p = 0.07). Other factors–sex, CD4^+^ count at enrollment, ART status–were not associated with protozoa infection. (See **Table 3**)

**Table 3:** Demographic and clinical factors associated with parasitic infection.

Demographic or Clinical Characteristic	n/N	Prevalence Ratio (95% CI)	n/N	Prevalence Ratio (95% CI)	n/N	Prevalence Ratio (95% CI)
***Univariable analyses***						
	Protozoa		Helminths		Hookworm spp.	
**Age, 5-year increments**	202	0.70 (0.54, 0.91)	202	1.12 (0.97, 1.30)	198	1.19 (1.01, 1.39)
**Women vs. Men**	8/139	0.73 (0.25, 2.13)	49/139	1.71 (1.00, 2.92)	45/137	1.82 (1.01, 3.28)
	5/63	—	13/63	—	11/61	—
**Weight, 5-kg increments**	196	0.94 (0.78, 1.13)	196	1.00 (0.90, 1.11)	192	0.99 (0.88, 1.10)
**WHO Stage 3,4 vs. WHO Stage 1,2**	2/25	1.24 (0.29, 5.27)	8/25	1.05 (0.56, 1.94)	6/24	0.87 (0.42, 1.81)
	11/170	—	52/170	—	48/167	—
**Currently receiving ART vs. ART-naïve**	12/181	1.39 (0.19, 10.23)	52/181	0.60 (0.36, 1.00)	46/179	0.49 (0.30, 0.80)
	1/21	—	10/21	—	10/19	—
**Farming occupation vs. Any other occupation**	11/131	2.90 (0.66, 12.75)	44/131	1.36 (0.84, 2.20)	40/130	1.35 (0.81, 2.27)
	2/69	—	17/69	—	15/66	—
	**N**	**Prevalence Ratio (95% CI)**	**N**	**Prevalence Ratio (95% CI)**	**N**	**Prevalence Ratio (95% CI)**
***Multivariable analyses***						
	Protozoa		Helminths		Hookworm spp.	
Age, 5 year increments^*^	202	0.71 (0.55, 0.92)	202	1.12 (0.97, 1.32)	198	1.20 (1.01, 1.42)
Women^**^	202	0.78 (0.27, 2.27)	202	1.71 (1.00, 2.91)	198	1.82 (1.10, 3.24)
Currently receiving ART^***^	202	1.00 (0.11, 9.41)	202	0.66 (0.41, 1.07)	198	0.57 (0.36, 0.93)
Farming occupation^***^	200	3.96 (0.89, 17.60)	200	1.28 (0.76, 2.14)	196	1.21 (0.68, 2.15)

* Sex-adjusted generalized linear model estimating prevalence ratios.

** Age-adjusted generalized linear model estimating prevalence ratios.

*** Age- and sex-adjusted generalized linear model estimating prevalence ratios.

### Factors associated with helminth infection and hookworm infection

There were no statistically significant associations between demographic and clinical characteristics and any helminth infection, i.e. either *A*. *lumbricoides*, *A*. *duodenale*, *N*. *americanus*, *S*. *stercoralis*, or *T*. *trichiura* from univariable analyses. Adjusting sex and/or age in multivariable models did not change these results. There were no statistically significant relationships between age, sex, occupation and ART status and prevalent helminth infection. Helminth infection was slightly more prevalent in women (PR = 1.71; 95%CI: 1.00, 2.91) and less prevalent in people currently receiving ART (PR = 0.66; 95%CI: 0.41, 1.07), but neither were statistically significant. (**Table 3**.)

Prevalent infection with hookworm species *A*. *duodenale* or *N*. *americanus* was positively associated with age, and female sex in univariable analyses. Current receipt of ART was inversely associated with prevalent hookworm infection. These relationships were exaggerated in multivariable models that adjusted for sex and/or age. Increasing age (PR_5-years_ = 1.20; 95%CI: 1.01, 1.42; p = 0.04) was associated with hookworm infection. Women were more likely to be infected with hookworm (PR = 1.82; 95%CI: 1.10, 3.24; p = 0.04), even after adjustment for age, versus male participants. Participants receiving ART were less likely to have prevalent hookworm infection (PR = 0.57; 95%CI: 0.36, 0.93; p = 0.02); 52.6% (10/19) of participants not receiving ART were infected with hookworm, and 25.7% (46/179) of participants receiving ART were infected with hookworm.

### Relationship between hookworm and immune status

We assessed the relationship between hookworm infection and CD4^+^ T helper cell concentrations at study enrollment. Participants with hookworm infection demonstrated consistently lower concentrations of CD4^+^ cells/mcL when compared to hookworm-uninfected peers (Table 4). Unadjusted analyses indicated an average difference of -70 cells/mcL (95%CI: -113, -26, p = 0.002) in participants with hookworm infected relative to those without detectable hookworm infection. This relationship became more pronounced when adjusting for participant age, sex, and time on ART; participants with hookworm infection had 94 fewer CD4^+^ cells/mcL on average (95%CI -133, -55, p = <0.001) than those without hookworm. Stratified analyses on ART status (receiving or not currently receiving ART) indicate a similar relationship among those persons receiving ART at enrollment (n = 171) (mean: -102 cells/mcL; 95%CI -145, -58; p = <001). An additional stratified analysis among those persons who were ART naïve was limited by a small sample size (n = 19), but did not show a statistically significant relationship between hookworm infection and CD4^+^ T cell concentrations (mean: -43 cells/mcL; 95%CI: -118, 32; p = 0.24).

**Table 4:** Differences in CD4+ cells/mcL between parasite infected- and parasite-uninfected adults living with HIV in peri-urban Uganda.

	N	Mean difference in CD4^+^ cells/mcL (95% CI) ^a^	p-value
**All participants**			
**Protozoa**			
**Protozoal infection, unadjusted**	202	-11 (-96, 75)	0.81
**Protozoal infection, adjusted** ^**b**^	194	-23 (-118, 71)	0.63
**Helminth**			
**Any helminth, unadjusted**		-62 (-107, -17)	<0.01
**Any helminth, adjusted** ^c^	194	-80 (-121, -39)	<0.001
**Hookworm**			
**Hookworm infection, unadjusted**	198	-70 (-113, -26)	0.002
**Hookworm infection, adjusted** ^c^	190	-94 (-133, -55)	<0.001
**Among ART initiated only**			
**Hookworm infection, adjusted** ^c^	171	-102 (-145, -58)	<0.001
**Among ART naïve only**			
**Hookworm infection, adjusted** ^d^	19	-43 (-118, 32)	0.24

^a^ Relative difference in CD4^+^ cells/mcL in those with hookworm infection, relative to those without hookworm infection.

^b^ Adjusted for nadir CD4, age, sex

^c^ Adjusted for nadir CD4, sex, years on ART

^d^ Adjusted for nadir CD4, sex

**Fig 1:**
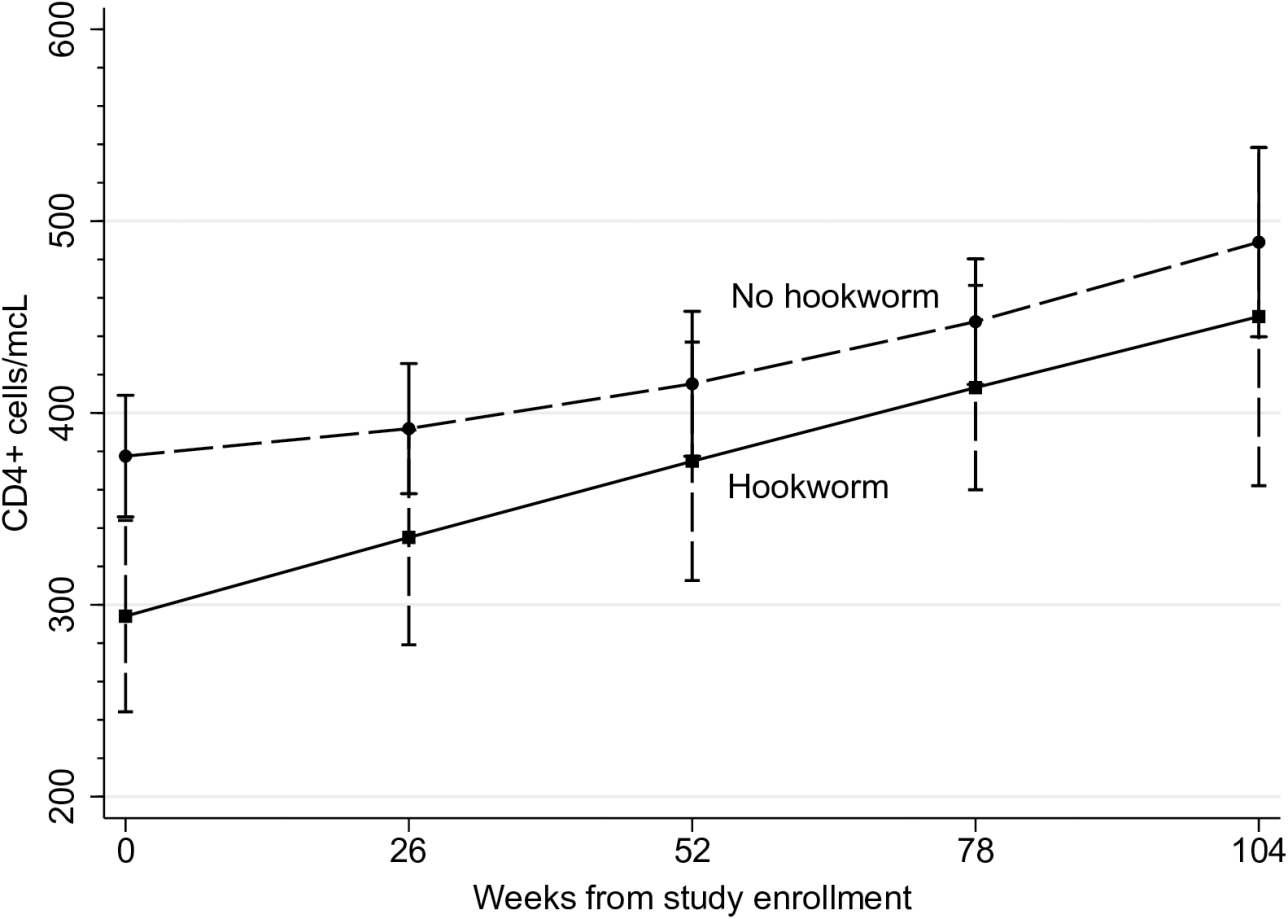
Change in CD4+ cells/mcL by hookworm infection status. CD4+ cells/mcL from baseline through 24 months of follow-up among participants infected with hookworm, versus those without hookworm infection enrolled at The AIDS Support Organisation (TASO), Mbale, Uganda.

Among participants who had initiated ART at enrollment, results from the longitudinal analyses demonstrate that participants with hookworm infection and participants without hookworm infection had a similar rate of CD4^+^ T cell immune recovery in the 24-months post-enrollment (β_hookworm-time_ = 0.55; 95%CI: -0.35, 1.45; hookworm-time interaction term p-value = 0.23). Participants with hookworm did, however, have consistently lower CD4^+^ concentrations relative to their hookworm-uninfected peers over the 24 months of follow-up (-87 cells/mcL; 95%CI -151, -22; p = 0.009), based on an average of 2.4 CD4^+^ measurements (min = 1, max = 5) (**Fig 1**). The mean number of measurements over time across hookworm-infected versus uninfected participants was similar (2.2 and 2.4, respectively).

At 12 months post-enrollment, predicted means from sex-, age-, time on ART- and baseline weight-adjusted linear mixed models estimated that participants with hookworm infection had an average of 361 CD4^+^ cells/mcL (95%CI 310, 412) versus an average of 419 cells/mcL (95%CI 391, 448) amongst those without hookworm infection at baseline. Participants with hookworm infection at baseline had, on average, 58 fewer CD4+ cells/mcL (95%CI: -117, 1) relative to their uninfected peers at 12-months of follow-up. At 24 months post-enrollment, participants with hookworm had predicted mean 438 CD4^+^ cells/mcL (95%CI 365, 514) versus mean 469 cells/mcL (95%CI 429, 509) among those uninfected with hookworm at baseline. While participants with hookworm infection still had lower CD4+ cell concentrations than their uninfected peers at 24 months of follow-up, the difference in average CD4+ cells/mcL between hookworm infected versus uninfected across groups was attenuated (-30 cells/mcL; 95%CI -114, 55) and not statistically significant.

Furthermore, participants who had initiated ART ≥1 year prior to study enrollment (n = 108) demonstrated a similar relationship to our overall cohort; there was no difference in rate of CD4+ cell recovery during the study period, but those with hookworm were at an immunologic deficit relative to their uninfected peers (77 fewer CD4+ cells/mcL in those with hookworm versus those without; 95%CI -154, -1). Among persons who had initiated ART <1 year prior to study enrollment (n = 68), the effect was less pronounced, with only 26 (95%CI: -121, 70) fewer CD4 cells/mcL in those with hookworm versus those without, and no difference in change over time, like other analyses.

## Discussion

We demonstrate that parasitic infection, particularly with *N*. *americanus* hookworm species, was common in this adult, HIV-infected population in Uganda. While these infections were generally light intensity infections, we report a clinically and statistically significant association between hookworm infection and decreased CD4^+^ T helper cells/mcL at study enrollment. This relationship was maintained over study follow-up, where participants with hookworm infection had diminished CD4^+^ immune status over time, relative to their peers who were not infected with hookworm. There was no difference, however, in CD4^+^ cell recovery over 24 months among participants who were ART-initiated at baseline.

To our knowledge, only two other studies to date have examined the health impacts of helminth infection in persons receiving ART, specifically the effects of deworming.[14, 15] In Uganda, Lankowski *et al*. did not find any significant beneficial effects of deworming in their overall study population. However, in a sub-group analysis of women only, they found that deworming with either albendazole or mebendazole 7 to 90 days prior to CD4^+^ T cell measurement, for unspecified parasitic infection increased CD4^+^ T helper cell concentrations by an average of 63 cells/mcL (95% CI: 6–120) in in the first year of ART initiation. This study was limited by the fact that a medical record of deworming was used as a proxy for helminth infection, and as such neither helminth infection prevalence nor deworming incidence were reliably captured. This may have attenuated their results towards a null finding in the overall cohort.

Ivan *et al*. found that deworming decreased HIV viral loads and increased CD4^**+**^ T cell concentrations over a 12-week period in a cohort of 980 HIV-infected, ART-initiated pregnant Rwandans.[15] While the results of this study demonstrate the value in revisiting the question of deworming in the presence of increasingly available ART, it is potentially limited by a treatment cross-over effect of deworming outside of the study setting. That said, any extra-study deworming in the control arm would likely attenuate the effects of their intervention; and one can extrapolate that the results in a completely controlled setting would have been more extreme. Additionally, this is a limited subset of the ART-receiving, HIV-infected population, and it would be important to duplicate these results in other populations of men and non-pregnant women.

### Prevalence of parasitic infection

Our results are comparable with much of the available literature regarding parasite infection prevalence in adults. Other studies conducted in Uganda have found similar prevalences of hookworm infection (24 to 52%),[35–39] and *Strongyloides* (4 to 8%) [40–42] in adults with and without HIV. In persons with HIV in Nigeria, Senegal, and Ethiopia, *Giardia* prevalence has been observed at approximately 5%.[43–45]

Partial immunity to most parasitic infections is acquired over the life course, leading to an increased rate of parasite destruction and worm expulsion with increasing age and re-infection. Hookworm species, however, do not induce the same adaptive immunity in humans as the other soil transmitted helminths, and consequently, may continue to infect adults with high frequency and intensity.[46] In the context of frequent and repeated infection, this lack of adaptive immunity may have important implications for host response to co-infections like HIV, and important Th1-moderated HIV co-morbidities, such as tuberculosis and cryptococcal meningitis.

### Relationship between hookworm infection and CD4^+^ T cells/mcL

Our results found that participants who were infected with hookworm were at a significant CD4^+^ T-helper cells/mcL deficit, relative to participants who were not infected with hookworm. CD4^+^ T-helper cells are critical in mediation of the immune system’s response to various pathogens, and commonly used to monitor HIV disease progression and response to ART.[47] The inverse relationship between hookworm infection and CD4^+^ T cell concentrations was qualitatively and statistically consistent across various analyses, from unadjusted to adjusted regression, analyses restricted to persons receiving ART, and over time. We did not observe a difference in CD4^+^ cells/mcL among persons who were ART-naïve; however, the small proportion of persons not receiving ART in this cohort (n = 19) renders these analyses relatively uninformative.

Ample evidence demonstrates that soil-transmitted helminths are potent immunomodulators, and infection with soil-transmitted helminths involves many major body systems, from the gastrointestinal and circulatory systems, to soft tissues.[19] Multiple biologic mechanisms could be driving our observed relationship; and these results are likely multifactorial for any given participant. Hookworm infection in HIV-uninfected persons with celiac disease has been shown to decrease expression of interferon (IFN)-γ on intestinal T cells, and increase in CD4^+^FoxP3^+^ regulatory T cells, which could contribute to decreased differentiation to CD4^+^ T helper cells.[48] Other research has demonstrated that hookworm antigens induce cytotoxic and pro-apoptotic activity in Jurkat T Cells, contributing to an increase in CD4^+^, CD8^+^, and CD19^+^ lymphocytes that were in an early and/or late stage of programmed cell death.[49] Cuellar *et al*. found that commonly excreted hookworm protein Ac-TMP-1, a Tissue Inhibitor of Metalloproteases, induced murine splenic T cells to differentiate to CD4^+^ and CD8^+^CD25^+^FoxP3^+^ regulatory T cells that expressed interleukin (IL-)10 and suppressed naïve and activated CD4^+^ T cells differentiation.[50] Other human studies, however, have not found similar increases in T regulatory responses to hookworm infection.[51]

Other human studies have not found differences in CD4^+^ T cell concentrations between hookworm-infected and -uninfected groups of HIV-uninfected participants. In a quasi-experimental study by George *et al*., which measured the impact of deworming on microbial translocation (a contributor to chronic immune activation linked to decreased concentrations of CD4^+^ T helper cells), observed that hookworm was associated with elevated levels of pro-inflammatory markers, e.g. lipopolysaccharide, soluble CD14.[52] They did not, however, observe differences in T cell subsets among naturally infected, HIV-uninfected participants at baseline.[52] The authors postulate that lack of difference in T cell subsets is mediated by a counterbalancing, anti-inflammatory effect of hookworm infection, e.g. elevated levels of IL-10, and decreased C-reactive protein, IL-17 and haptoglobin.[52]

From the standpoint of clinical endpoints, results from clinical trials conducted in ART-naïve persons remain mixed. Results from the HEAT trial, which evaluated the impact of reflexive and repeated deworming on a patient’s risk for ART eligibility, i.e. a drop below 350 CD4^+^ cells/mcL, found no difference between the reflexive deworming group (400 mg albendazole every 3 months plus 25 mg/kg praziquantel annually) versus the standard of care group (no empiric deworming).[10] The trial had 80% power to detect a hazard ratio of 0.775, which could be considered a large, albeit clinically important, difference in treatment groups. That said, actual CD4^+^ T cell concentrations at study completion were very similar across randomization groups, supporting the idea that deworming may not dampen CD4^+^ decline in the absence of ART. Other studies support this conclusion.[4, 5, 53] However, still other studies and meta-analyses demonstrated reductions in plasma viral loads and increases in CD4^+^ T helper cells with deworming in persons living with HIV.[13, 54] The differences in these results may in part be explained by differences in methodology, and in particular the need to pool species due to limited species-specific sample sizes.

Repeated and long-term exposure to hookworm and other helminth species may cause fibrosis of the gut associated lymphatic tissues (GALT). IL-13, in particular, is increased in the presence of hookworm infection[46] and a dominant mediator of fibrotic tissue, which induces fibrosis independently and via simulation and activation of transforming growth factor (TGF)-β. In the case of chronic and repeated helminth infections, and corresponding Th2 type immune responses, IL-13 production can become pathological. Fibrosis of the GALT has been linked to the dysregulation of immune cells, including CD4^+^ T cells, and impaired CD4^+^ recovery.[55, 56]

Chronic and repeated exposure to helminths and subsequent GALT fibrosis may have impacted the results of this and other studies. Indeed, the longitudinal analyses in this study demonstrate that hookworm-infected versus uninfected participants have a similar rate of CD4^+^ recovery over the 24-month follow-up period, but that those infected with hookworm remained at a significant immunologic deficit relative to their uninfected peers over time. Fibrosis is not reversible with deworming or other therapy. This bears mentioning because while hookworm and other helminths are still causally implicated in the decrease in CD4^+^ T cell concentrations, there are important implications for public health intervention design, e.g. increased deworming frequency targeting all stages of the human life course.

### Limitations

It is possible that our results are spurious, either due to confounding, a misunderstanding of the directionality of the hookworm-CD4^**+**^ relationship, or a type I statistical error. The primary limitations of this study arise from its observational nature. First, the temporal relationship between parasite infection and immune status remains undetermined; it is conceivable that being immunocompromised would increase the likelihood of persistent infection. Research on this topic remains mixed and parasite dependent.[57–60] However, most research to date suggests no difference in hookworm risk between immunocompromised and immunocompetent persons.[61–64] There is potentially one exception to this pattern. A cross-sectional study by Sanyaolu *et al*. found that 4.6% (3/65) of HIV-infected Nigerians had hookworm infection, versus 1.8% (18/1015) of HIV-uninfected peers.[65] However, we were unable to duplicate their results based on the data provided in the paper. Our results are based on a single stool sample, and we did not use any concentration techniques prior to DNA extraction. Diagnostic sensitivity for hookworm–and other species–when evaluating a single stool sample is lower than sensitivity when using multiple stool samples. For example, Knopp *et al*. found that a single stool sample yielded a 7.1% prevalence, while 2 samples yielded a prevalence of 15.6% via Kato-Katz.[66] These authors found that *Strongyloides* prevalence with 1 stool sample versus 2 samples was similar, 3.5% and 5.3%, respectively. It is unlikely that our observed hookworm prevalence would have doubled had we analyzed >1 stool sample. However, we may have misclassified parasite-infected participants as uninfected, particularly among those with a low burden.

Finally, our results may be confounded by data that would have been useful in these analyses but were not available. Hookworm, and other intestinal parasites, are considered diseases of poverty. The relationship between increased infection incidence among economically disadvantaged persons is well established.[38, 67–69] Additionally, being economically disadvantaged could have impacted health outcomes in this study, e.g. CD4^+^ T helper cells concentrations, as it has in other research.[70–72] While we collected information on place of residence, this information could ultimately only be dichotomized into participants who lived in Mbale town, where the clinic is located, versus all others, which could represent varied levels of development and corresponding hookworm exposure. Also, ART adherence data were routinely collected and reflected uniformly high adherence levels. Past research on adherence at TASO ART clinics report similarly high levels of ART adherence, with ~90% of patients reporting no missed pills in the past 30 days.[73–75] However, data from prior TASO adherence research–like adherence data for this study–are limited by the fact that they are self-reported, which consistently over-reports adherence relative to pill counts, pharmacy refill information, and/or drug concentrations in blood.[76, 77]

## Conclusions

Despite the limitations of this study, we feel that these results are generalizable to other adults receiving outpatient HIV therapy in low-income, peri-urban areas. The results presented herein point to a high prevalence of helminth infection in this vulnerable population, and that hookworm infection is associated with sub-optimal health outcomes, i.e. lower CD4. Therefore, further examination of these questions via a randomized trial is warranted, especially how systematic deworming may impact the immune status of this vulnerable population in the presence of ART.

## Supporting information

S1 STROBE (DOCX)

S1 DNA Extraction Protocol (DOCX)

S1 Data (XLSX)
