# Supplementary material for: Hookworm infection is associated with decreased CD4+ T cell counts in HIV-infected adult Ugandans
Source: PLoS Negl Trop Dis. 2017 May 25;11(5):e0005634. doi: 10.1371/journal.pntd.0005634 (PMC5462474; doi:10.1371/journal.pntd.0005634)
Supplement: S1 DNA Extraction Protocol — (DOCX) [file pntd.0005634.s002.docx]

# S1 DNA Extraction Protocol

# Low-reagent DNA Extraction Protocol using the MP Biomedicals FastDNA SPIN Kit for Soil

# Add 978 µl Sodium Phosphate buffer to Lysing Matrix E tube.

# Use same tip to load all samples

# Add 122 µl MT Buffer.

# Use same tip to load all samples

# Add 50 mg of stool to each tube.

# Use new toothpick or new pipet tip to load

# Homogenize

# FastPrep Instrument 40 sec on speed setting 6.0

# Mini Beadbeater 2 min

# Disruptor Genie 5 min 3000 rpm

# Centrifuge 14,000 g for 10 min

# Add 250 µl PPS to new 2 ml tube

# Use same tip

# Pipet supernatant from (Step 5) into 2 ml tube (Step 6) and invert 10 times by hand

# Centrifuge 14,000 g for 10 min

# Add 2µl Internal control (PBr322, 10^4^), E. coli plasmid

# Do not MIX

# In 2 new tubes, add 500 µl binding matrix

# Re-suspend binding matrix vigorously and pipet from bottom of bottle

# Use same tip to load binding matrix

# Mix binding matrix every 10 tubes

# Add supernatant from step 9 approximately 600 µl to each tube

# Volumes may vary, divide the supernatant equally

# Use same tip per sample, but change between numbers

# Invert for 2 min (Can store at 4 C for 1 hour at this step.)

# Let sit for 5 min at room temperature.

# Remove almost all supernatant from each tube.

# Remove at angle, careful not to disrupt matrix

# Remove enough supernatant leaving approximately 1 mm of volume behind

# Use same tip per sample, but change between numbers

# Remove entire binding matrix

# Use same tip from Step 14

# Add to spin filter with tube

# Centrifuge 14,000 g for 2 minutes.

# Depending on consistency of sample, more spins maybe needed to elute fluids

# Empty catch tube and discard fluid

# Add 500 µl of PREPARED SEWS-M to matrix and mix with gentle pipetting.

# Careful not to pierce membrane at bottom of filter tube

# Centrifuge 14,000 g for 2 min

# Empty catch tube and discard fluid

# Centrifuge 14,000 g for 2 min to help dry the matrix.

# Replace the tube with final catch tube.

# Air dry the filter for 5 min at room temp

# Leave lid open

# Add 100 µl of DES and mix matrix with gentle STIRRING

# Centrifuge 14,000 g for 2 min

# Store sample at 4 C or -20 C for long term storage

#

# Trichuris extraction Trichuris extraction can be done at same time as above extraction. From Step 5 above:

# Add 200 µl H20 to used Lysing Matrix E tube

# Shake vigorously for 1 min

# Heat at 90 C for 10 min

# Shake vigorously for 1 min

# Centrifuge 14,000 g for 10 min

# Using the same tip, add 50 µl PPS to new 2 ml tube

# Can also use same tube from (Step 6 above) for the same patient sample

# Pipet supernatant from (Step 5) into 2 ml tube (Step 6) and invert 10 times by hand.

# Centrifuge 14,000 g for 10 min

# Add 2 µl Internal control (PBr322, 104)

# Do not MIX

# In 1 new tube, add 500 µl Binding matrix

# Re-suspend binding matrix vigorously and pipet from bottom of bottle

# Use same tip to load binding matrix

# Mix binding matrix every 10 tubes

# Add supernatant from step 9

# Invert for 2 min (Can store at 4 C for 1 hour at this step.)

# Let sit for 5 min at room temperature

# Remove supernatant and discard.

# Remove at angle, careful not to disrupt matrix

# Remove enough supernatant leaving approximately I mm of volume behind

# Use same tip per sample, but change between numbers

# Remove entire binding matrix

# Add to spin filter with tube

# Centrifuge 14,000 g for 2 min

# Depending on consistency of sample, more spins maybe needed to elute fluids

# Empty catch tube and discard fluid

# Add 500 µl of PREPARED SEWS-M to matrix and mix with gentle pipetting

# Careful not to pierce membrane at bottom of filter tube

# Centrifuge 14,000 g for 2 min

Empty catch tube and discard fluid

# Centrifuge 14,000 g for 2 min to help dry the matrix

# Replace tube with final catch tube

# Air dry the filter for 5 min at room temp with lid open

# Add 50 µl of DES and mix matrix with gentle STIRRING

# Centrifuge 14,000 g for 2 min

# Store sample at 4 C or -20 C for long term storage
